# Content analysis of conversations on Reddit: reactions to FDA’s ENDS prioritized enforcement policy

**DOI:** 10.3389/fcomm.2024.1348158

**Published:** 2024-09-16

**Authors:** Jamie Guillory, Sarah Trigger, Jenna Brophy, Ashley Ross, Stephanie Lane, Annice Kim, James Nonnemaker, Sherry T. Liu, Kimberly Snyder, Janine Delahanty

**Affiliations:** 1 RTI International, Dublin, Ireland,; 2 Office of Science, Center for Tobacco Products, Silver Spring, MD, United States,; 3 RTI International, Durham, NC, United States

**Keywords:** electronic nicotine delivery systems, policy, social media, tobacco, flavored tobacco, public policy

## Abstract

**Introduction:**

On January 2, 2020, the FDA announced a policy focused in part on prioritizing enforcement of flavored (other than tobacco- or menthol-flavored) cartridge-based electronic nicotine delivery systems (ENDS) without premarket authorization.

**Methods:**

We used a query to identify Reddit conversations relevant to the policy from January 2 to May 6, 2020. Our sample included 576 posts (46 posts and 530 accompanying comments). Two analysts coded posts for mentions of use behaviors (e.g., switching, quitting), purchasing behaviors (e.g., purchasing from retailer new to the user), and flavored products. We summarized frequencies of coded data and provided illustrative quotes.

**Results:**

Only 21.0% (121/576) of posts mentioned use behavior. Switching behavior was the most common use behavior mentioned (50.4%, 61/121). Most switching behavior posts focused on ENDS-related switching (91.8%, 56/61). The most common ENDS-related switching behaviors mentioned were switching to an open tank (45.9%, 28/61) or device with refillable pods/cartridges (44.3%, 27/61); 8.2% (5/61) mentioned switching to disposables. Just 15.5% (89/576) of posts mentioned purchasing behavior, with the most common being purchasing from a retailer new to the user (32.6%, 29/89). Only 6.8% (39/576) of posts mentioned specific flavors.

**Conclusion:**

Reddit posts about the policy commonly discussed switching to non-cartridge-based ENDS products, such as open tank systems or disposable devices, and purchasing products from different online sources that were still selling these products. Findings suggest that publicly available Reddit data can complement data from traditional sources (e.g., surveys, sales) to understand potential unintended consequences associated with policies by exploring the public’s reactions.

## 1 Introduction

The 2009 Family Smoking Prevention and Tobacco Control Act and subsequent 2016 “deeming rule” provided the U.S. Food and Drug Administration (FDA) authority to regulate the manufacture, distribution, and marketing of tobacco products, including electronic nicotine delivery systems (ENDS) (Food and Drug Administration, HHS, 2016). All deemed new tobacco products on the market as of August 8, 2016 were required to submit a premarket application by September 9, 2020. FDA maintains the ability to prioritize enforcement against any unauthorized new tobacco product.

On January 2, 2020 (revised April 2020), FDA issued final guidance to industry that announced a policy prioritizing, among other things, enforcement for some unauthorized flavored (e.g., fruit, mint) cartridge-based ENDS products that may appeal to young people (U.S. Food and Drug Administration, 2020a).^1^ Companies had 30 days to “cease the manufacture, distribution, and sale of unauthorized flavored cartridge-based e-cigarettes (other than tobacco or menthol)” or risk FDA enforcement actions when the enforcement policy went into effect on February 6, 2020.^2^

Several studies examined responses to the policy. After policy implementation, adult JUUL users reported less use of mint-flavored ENDS pods (explicitly prioritized for policy enforcement) and more use of menthol-flavored ENDS pods (explicitly not prioritized for policy enforcement) (Yingst et al., 2020). Another study showed sales of mint-flavored prefilled ENDS cartridges decreased following the policy (Ali et al., 2020; Diaz et al., 2020). Between August 2019 and May 2020, mint-flavored prefilled cartridge sales decreased from 47.6 to 0.3% of all prefilled cartridge unit sales, while menthol- and tobacco-flavored prefilled cartridge sales increased from 10.7 to 61.8% and 22.8 to 37.1% of all prefilled cartridge unit sales, respectively (Ali et al., 2020). In the 8 weeks following the policy announcement, U.S. ENDS market share of mint-flavored ENDS decreased 82.8% while share of menthol-flavored ENDS increased 104.9% (Diaz et al., 2020). Around the time of and following policy implementation, sales also increased for disposable ENDS that had greater product capacity and nicotine strength and lower cost per milliliter (Diaz et al., 2023). Relatedly, survey research suggests that young adult users of flavored ENDS most commonly responded to state and local restrictions on all flavored ENDS by continuing to vape (Tam et al., 2024).

While sales and survey data are important for understanding policy effects, social media data can provide complementary information on how people share and seek information around policies to provide early signals for how consumers may respond to policies, including in-depth, timely insights into how policy influences perceptions and tobacco use behaviors (e.g., initiation, use, substitution, cessation). The Comprehensive Model of Information Seeking suggests that antecedent factors such as personal experience with and salience of an issue to an individual influence the utility of information held by an information carrier and that utility along with characteristics of the information carrier influence information seeking actions (Johnson et al., 1995). Factors in this model apply to information seeking behavior around ENDS-related policies on Reddit. First, policies influencing the availability of ENDS products are salient to ENDS users and on Reddit people engage in rich, descriptive dialogue in self-organized communities of interest (e.g., communities focused on ENDS use) indicating utility to ENDS users seeking information around these policies. Further, Reddit as an information carrier provides opportunities to engage in in-depth dialogue due to no character maximum and the platform’s interactive nature making it useful for understanding perceptions and behaviors. While Reddit may be less popular among teens and adults than other platforms (e.g., YouTube) (Anderson and Jiang, 2018; Auxier and Anderson, 2021), Reddit insights may allow for in-depth understanding of attitudes and reported behaviors around ENDS use. Reddit users also have “karma” provided based on “upvotes” (i.e., user action signaling approval), which is a characteristic of individual information carriers within Reddit and serves as an indicator of users’ level of influence and popularity and increases the likelihood that users’ content will be viewed by information seekers.

Several studies have explored how users seek information about ENDS-related policies on Reddit. One article analyzed Reddit conversations about vaping policies (Xu et al., 2021). Another study (Silver et al., 2022) analyzed themes related to policy circumvention in Reddit conversations around ENDS restrictions, including the ENDS prioritized enforcement policy. Previous studies have also examined Reddit discussions about ENDS flavors (Wang et al., 2015; Zhan et al., 2017; Chen et al., 2020; Lu et al., 2020; Luo et al., 2021), ENDS use (Brett et al., 2019; Zhan et al., 2019), ENDS use motivations (Sharma et al., 2016), purchasing and selling ENDS (Liu et al., 2020), ENDS product reviews (Allem et al., 2019), e-liquid ingredients (Li et al., 2016), and topic analysis (Barker and Rohde, 2019; Hu et al., 2021; Xu et al., 2021).

This study investigated public response to the ENDS prioritized enforcement policy by analyzing Reddit conversations to qualitatively describe ENDS use, switching between products, and purchasing behaviors, which is crucial to understanding reactions to the policy and has yet to be explored with Reddit data. Results will also contribute to the growing body of research examining responses to the policy (Ali et al., 2020; Diaz et al., 2020; Yingst et al., 2020) by providing qualitative information to contextualize these findings.

## 2 Methods

This study was determined non-human subjects research by RTI International’s Institutional Review Board.

### 2.1 Relevant Reddit terms

On Reddit, users share content as a “post.” A “subreddit” is a group dedicated to one topic. A “comment” is a post by a user replying to an initial post or other comment. A “comment thread” involves one or more users responding to a post comment, becoming its own discussion; there may be multiple threads in reaction to one initial post. “Karma” is provided for each user and is based on “upvotes” (i.e., user action signaling approval that is similar to a “like”) (Reddit, 2021).

### 2.2 Data source and sample

**FIGURE 1:**
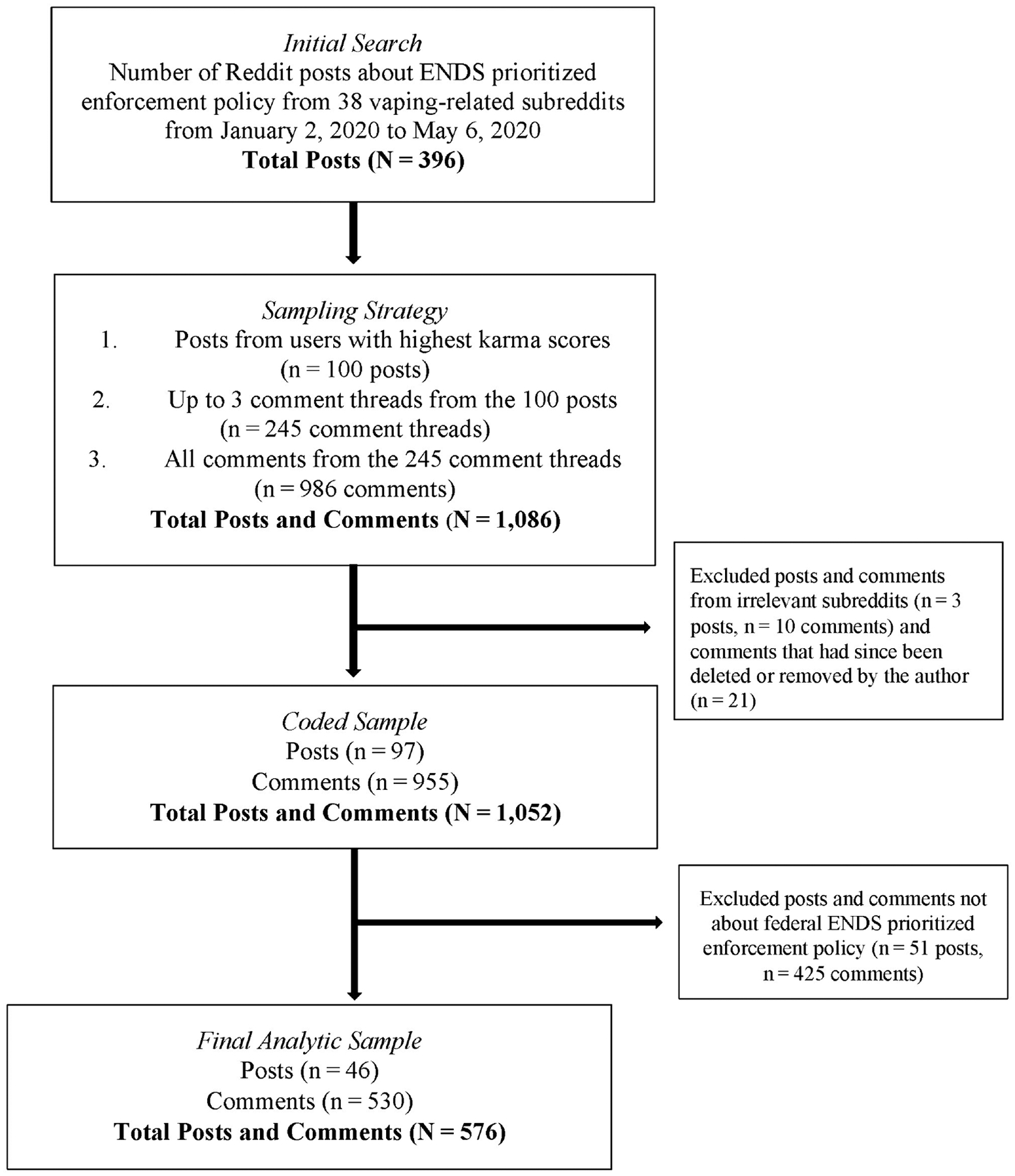
Sampling of Reddit posts and comments about FDA ENDS prioritized enforcement policy.

To identify relevant discussions, we searched for terms such as “flavor policy(–ies)” or “flavor(s) ban*” posted in subreddits with ENDS content from January 2 to May 6, 2020 (see Supplementary material for query and subreddits). We used the chosen search terms to explore conversations in ENDS-related subreddits to reduce irrelevant content. Posts were English-language only. Searches were conducted in Brandwatch, a platform that provides users access to social media data. We identified an initial sample of 396 posts (Figure 1). Data were collected and coded from September through November 2020.

To narrow the coding sample, we first selected posts published by users with the highest karma scores. We selected the top 100 posts based on karma scores, limiting one post per user to ensure the sample was not skewed by individual users’ perspectives. We prioritized posts based on the highest karma scores (Struik and Yang, 2021) to focus on popular posts with higher engagement.

For each of these 100 posts, we selected up to three comment threads with the highest number of upvotes to focus on comments with higher engagement. The number of comment threads in these posts ranged from zero to 42 (mean = 7; median = 4 [IQR: 2–8]). At this stage, we included all comments in selected threads for coding. This resulted in an initial sample of 100 posts with 986 comments from 245 comment threads.

After the initial prioritization, we excluded irrelevant posts and accompanying comments (e.g., focused on conversations outside the U.S.) (3 posts, 10 comments) and excluded posts or comments that the author deleted (0 posts, 21 comments). We coded the resulting sample of 97 posts and 955 comments.

To ensure our sample focused on conversations related to the federal ENDS prioritized enforcement policy, we removed individual posts and comments that discussed only state or local policies or did not reference the federal policy (51 posts, 425 comments). For example, if a post discussed a state-level policy it was excluded, but if accompanying comments discussed the federal policy those were included.

Our final analytic sample included 46 posts and 530 comments (Figure 1). Each post and comment were coded separately and treated as equivalent units of analysis, for an analytic sample of 576 posts and comments from 282 unique authors. Posts and comments are hereafter referred to as posts for brevity.

### 2.3 Coding

We developed a codebook to capture themes about use and purchasing behaviors and mentions of ENDS flavors. Themes were not mutually exclusive, meaning a post could be coded for multiple themes. Posts included discussion of anticipated, actual, or recommended behaviors (e.g., predicting other users’ behaviors in response to the policy). All posts in the sample were double coded by two trained coders using qualitative coding software, NVivo 12. A third adjudicator independently resolved discrepancies.

#### 2.3.1 Use behaviors

Use behaviors were coded when Reddit users discussed behaviors related to using ENDS or other tobacco products in response to the policy.

Switching behaviors were coded when users discussed products they were switching to in response to the policy (e.g., other ENDS device types, other tobacco products). ENDS-related switching included: (1) switching to open tank devices (e.g., tank systems); (2) switching to devices with refillable pods or cartridges (explicitly described as refillable) that can be filled with one’s choice of e-liquid; (3) switching to disposable devices sold ready for use (prefilled and charged); (4) switching to prefilled cartridge-based ENDS (device and pods) with tobacco- or menthol-flavored pods/cartridges; (5) switching to zero-nicotine e-liquids; and (6) other ENDS-related switching. We also coded for switching behaviors related to other products, including switching to non-ENDS tobacco products and cannabis.

Other use behaviors coded were: (1) mixing own e-liquid (i.e., do-it-yourself [DIY]); (2) cutting back or quitting ENDS; (3) continued use of flavored (non-menthol and non-tobacco) cartridge-based ENDS (includes cartridges/pods sold separately); (4) other ENDS use behaviors; and (5) other tobacco use behaviors.

#### 2.3.2 Purchasing behavior

Purchasing behaviors in response to the policy were coded as: (1) purchasing from a retailer new to the user; (2) purchasing from a non-U.S. jurisdiction; (3) purchasing extra (i.e., stockpiling) ENDS not mentioned in the policy; (4) purchasing extra (i.e., stockpiling) flavored (non-menthol and non-tobacco) cartridge-based ENDS with pods/cartridges; (5) purchasing from another user or noncommercial source; and (6) other purchasing behavior.

#### 2.3.3 Flavor coding

Flavor was coded when a flavor was mentioned in relation to ENDS as follows: (1) flavored products included in the policy (i.e., flavored [other than menthol- or tobacco-flavored] pod/cartridge-based ENDS, including devices sold with cartridges/pods and cartridges/pods sold separately); (2) all other flavored products not included in that policy provision (i.e., all flavors of non-cartridge-based e-liquid, menthol- or tobacco-flavored pods, all flavors of disposable devices); and (3) other flavored ENDS, which included cases where a specific flavor was mentioned but device type was unclear.

### 2.4 Analysis

We summarized frequencies of coded themes and provided illustrative post examples. To protect privacy, we paraphrased examples or used partial quotes. We also described instances where behavior themes intersected with flavored product mentions.

## 3 Results

### 3.1 Use behavior

Twenty-one percent of total posts mentioned use, including switching and other use behaviors (Table 1).

#### 3.1.1 Switching behavior

Among posts mentioning use behavior, switching was most common (50.4%). Within switching, ENDS-related switching was most common (91.8% of switching behaviors in posts). Within ENDS-related switching, discussion of switching to an open tank (45.9%) or refillable device (44.3%) was most common. Users often said they planned to switch devices or products in response to the policy and requested or provided recommendations. Users were occasionally in search of fruit- or mint-flavored products to replace previous cartridge-based ENDS. In a post about switching to an open tank, one user asked for recommendations to replace “*Iced Mango*” Zpods.

Switching to a disposable device appeared in 8.2% of switching posts: users posited that many would switch to disposables or discussed switching themselves. One user said that many teens had already switched from JUUL to disposable Puff Bar “*to get good [flavors]*” without maintaining a tank device. Mentions of switching to cartridge-based ENDS with tobacco- or menthol-flavored pods/cartridges (4.9%) were uncommon. Users occasionally described trying, but not liking, these flavors. Mentions of switching to zero-nicotine e-liquid were uncommon (1.6%).

Mentions of switching to other non-ENDS tobacco products (6.6%) and cannabis (1.6%) were uncommon. Users occasionally noted other people would begin smoking cigarettes again following the policy or that they themselves would smoke or dip.

#### 3.1.2 Other use behaviors

The second most common use behavior was mixing e-liquid (i.e., DIY activities) (24.0% of use behavior posts). One user said they blended e-liquids (e.g., “*mix ‘hot cocoa’ and ‘almond’*”). Another user noted DIY is “*easy and cheap*” and others should try it; another user noted that there are “*apps with hundreds of [DIY] recipes*.”

Cutting back or quitting ENDS was discussed in 12.4% of use behavior posts. Users occasionally discussed how the policy resulted in cutting back or quitting ENDS, which some said would be beneficial because the habit was “*expensive*.” Users occasionally discussed quitting for health reasons; one noted ENDS were “*taking my life away*.”

Discussion of continued use of flavored (other than tobacco- or menthol-flavored) cartridge-based ENDS occurred in 8.3% of posts. Posts often discussed policy circumvention. One user said teens would “*always find a way*” to continue using restricted products via overseas or black market sources. Users occasionally mentioned using restricted flavors; one said they now use fruit-flavored “*knock off pods… and hate it*.”

Other ENDS use behaviors (i.e., use behaviors not in pre-defined themes) were discussed in 8.3% of posts. Users asked questions about the policy; mentioned stockpiling unrestricted products (e.g., nicotine e-liquids, coils) in preparation for potential new policies (e.g., advising freezing nicotine e-liquids for long-term storage); or noted general ENDS use. One user wanted to know whether the menthol flavor changed after the policy, saying that the menthol-flavored cartridges tasted different to them and they might seek alternatives.

Discussion of other tobacco use behaviors with ENDS use (i.e., dual use) was infrequent (5.8%). These posts often overlapped with switching to non-ENDS tobacco products. One user said they will continue using smokeless if “*flavors do get banned*” in ENDS.

### 3.2 Purchasing behavior

Overall, 15.5% of posts mentioned purchasing behavior (Table 2). The most frequent purchasing behavior was purchasing from a retailer new to the user (32.6%), including specifying retailers they were currently purchasing from or planned to purchase from or requesting suggestions (e.g., “wevapeusa.com,” “*pricepointny*”). One user noted “*discounthookahandvape*” sold policy-restricted mango- and mint-flavored cartridges.

In 21.3% of purchasing posts, users mentioned purchasing products prioritized for enforcement from non-U.S. jurisdictions (e.g., Canada). Users asked for source recommendations; in response, users noted retailers outside the U.S. selling restricted products to U.S. customers.

We identified purchasing extra other ENDS products (i.e., products not included in the policy) in 21.3% of purchasing posts. Users noted concerns about supply, and suggested stocking up on nicotine, e-liquid, coils, and devices (e.g., open/refillable devices). One user stated that users will regret not stockpiling products containing nicotine assuming these products could be taxed.

Purchasing extra flavored (other than menthol- and tobacco-flavored) cartridge-based ENDS (device and pods) was mentioned in 13.5% of purchasing posts. Users encouraged product stockpiling (“*find a flavor you enjoy and stock up*”) as JUUL’s flavored cartridge production stopped. One user doubted the policy would go through but advised others to stock up. No mentions of purchasing from a direct source (e.g., another user or non-commercial source) were observed.

Other purchasing behaviors were common (36.0%), including discussing features of devices users purchased or recommended purchasing, general purchase behavior mentions, or purchasing normal quantities (i.e., not stockpiling) of unrestricted products (e.g., nicotine, mod parts). One user preferred tobacco flavors, and reported being unaffected by the policy.

### 3.3 Specific flavors

Table 3 describes specific flavor mentions (e.g., flavored, menthol, tobacco) within use and purchasing posts. Flavor mentions were infrequent (6.8% of total). Specific flavors were mentioned in 24.8% of use behavior posts (5.2% of total), 27.9% of switching behavior posts (3.0% of total), and in 16.9% of purchasing behavior posts (2.6% of total). Posts about use and purchasing that mentioned flavors often discussed previously described themes.

New themes emerged in posts mentioning specific flavors. One theme centered around seeking to clarify what flavors and devices the policy affected. One user asked whether fruit flavors of “*Flair Envy*” or “*Cali*” devices would remain available. Another user considered switching to refillable pods, asking if they were “*still going to be legal*.” A second theme involved asking for or providing flavor recommendations to replace restricted products. One user requested mint-flavored e-liquid recommendations; others recommended “*Pod juice Jewel Mint*,” “*Jewel Mint Sapphire*,” and “*Arctic Air*.” A third theme was users experimenting with, but disliking, new flavors. One user responded to those who recommended a brand of honeydew, stating “*it was worse”* than their previous honeydew flavor.

## 4 Discussion

### 4.1 Summary of findings

We analyzed Reddit conversations describing use and purchase behaviors in the 4 months following FDA’s announcement of a policy prioritizing enforcement of flavored (other than tobacco- and menthol-flavored) cartridge-based ENDS. Users most frequently talked about switching to other ENDS that were not the focus of this provision of the policy (i.e., open/refillable systems). This finding is consistent with Silver et al. (2022) research showing that consumers on Reddit discussed substitute products (e.g., disposable or refillable devices) in discussions around ENDS flavor policies, including the prioritized enforcement policy. Recent studies analyzed ENDS sales data after the policy and found increases in sales of ENDS products that were not the focus of the policy, including (1) sales of flavored (other than tobacco- or menthol-flavored) disposable products, menthol- and tobacco-flavored prefilled cartridges (Ali et al., 2020; Diaz et al., 2020) and (2) sales of all menthol-flavored ENDS (Ali et al., 2020; Diaz et al., 2020).

Users posted less frequently about switching to other ENDS not explicitly prioritized by the policy (e.g., cartridge-based devices with tobacco- or menthol-flavored pods or cartridges, any flavor disposable devices). This finding was a bit surprising, given that disposable devices are available in many flavors (Ramamurthi et al., 2023), but this may reflect the sample of Reddit users. Although we are unaware of data characterizing ENDS users posting on Reddit, 18% of U.S. adults aged 18 and older (36% aged 18–29; Auxier and Anderson, 2021) and 7% of youth aged 13–17 report Reddit use (Anderson and Jiang, 2018) while research shows that disposable ENDS are more popular among youth than young adults and adults (McCauley et al., 2022). Few users in our sample discussed switching to non-ENDS tobacco products (e.g., cigarettes) or cannabis.

After switching, the second most common use behavior mentioned was mixing one’s own liquids, several of which emanated from the r/DIY_eJuice subreddit (14%; 4/29), and potentially suggesting attempts to mimic restricted flavors. Fewer users discussed cutting back or quitting ENDS in relation to the policy.

In conversations about purchasing behaviors, users mentioned purchasing from a retailer they had not previously purchased from (e.g., specific online retailers), purchasing products from outside the U.S., and stockpiling products in anticipation of the policy. Silver et al. (2022) similarly showed that consumers on Reddit discussed purchasing products online from outside the U.S. in discussions around ENDS flavor policies, including the prioritized enforcement policy. Research shows that more comprehensive flavored tobacco product restrictions (i.e., policies that do not exempt certain product categories, flavors, or retailers) can help reduce unintended consumer responses, such as switching to exempt products or purchasing from alternative retailers observed here (Gammon et al., 2022). The most common purchasing-related mentions were not in a pre-defined (i.e., *a priori*) coding category, including discussions of device features, purchase recommendations, and general purchase behaviors.

Specific flavors were mentioned infrequently. Many posts mentioning flavor discussed continued use of or purchasing extra flavored (other than tobacco- or menthol-flavored) cartridge-based ENDS. Other themes around flavors included asking questions about or clarifying flavors/devices affected by the policy; asking for or providing flavor recommendations to replace restricted products; and trying, but disliking, new flavors as a result of the policy.

These qualitative analyses of Reddit data provide more detailed insights into the range of use and purchasing behaviors in response to the ENDS prioritized enforcement policy than traditional public health surveillance sources (e.g., surveys, sales data) and suggest that social media data can provide meaningful insights into how people gather and share information about behaviors associated with health policies. A previous study of Instagram and Twitter complements our findings, showing ENDS brands were not using Instagram and Twitter to market cartridge-based ENDS devices with any tobacco, menthol, or other flavor, but were advertising devices without mentioning flavor (e.g., open/refillable, disposable) (Guillory et al., 2024). Social media data, being available in real time, have the potential to reveal timely reactions to policies across many products and retailers, which may not be possible with other data sources. Recent studies have demonstrated the utility of using social media data to assess public perceptions of policies, for example illustrating Reddit users’ discussion of ENDS flavor policy circumvention (Silver et al., 2022), showing that Twitter users’ sentiment around ENDS changed (Lu et al., 2022) and that number of mentions about quitting vaping increased after the announcement of the enforcement policy (Xie et al., 2022).

Our findings highlight the usefulness of conducting ongoing monitoring of social media and other data sources, such as the approach employed by the Center for Rapid Surveillance of Tobacco (CRST, 2024) to monitor tobacco industry marketing and emerging products, to anticipate responses and potential adaptations to future tobacco policy changes. Such monitoring could inform messaging efforts on cessation or to counteract misinformation. It is important to acknowledge that while Reddit data can provide signals for surveillance and act as an information resource for users around policy events, it may provide an incomplete picture of policy responses and should be considered alongside signals from other data sources, including sales and surveys. For example, there were few mentions of switching to flavored (other than tobacco- or menthol-flavored) disposable products and menthol- and tobacco-flavored prefilled cartridges despite sales of these products increasing around this time period (Ali et al., 2020; Diaz et al., 2020). However, these studies did not capture sales of open/refillable devices, which suggests that Reddit data can fill a gap by highlighting responses related to products not captured in sales data.

### 4.2 Limitations

This study has several limitations. This study focuses on conversations among a limited sample of Reddit posts based on the query used and sampling method. Furthermore, Reddit users represent a subset of the population that is actively, publicly discussing and sharing information about ENDS. Thus, findings may not generalize to conversations among all Reddit users, or to users on other platforms. We also do not know the characteristics of Reddit users who posted about the policy (e.g., age, ENDS use status). We collected posts from September through November 2020 for a retrospective time period of January through May 2020; users may have deleted or altered posts between the time of posting and data collection and we acknowledge the age of the data in the present study as a limitation. The study period included the final policy announcement (January 2, 2020) and the start of the policy (February 6, 2020); conversations may differ between the announcement period (policy anticipation) and the policy implementation. This study did not examine conversations around the November 2018 initial statement by the FDA Commissioner detailing aspects of the guidance, the March 2019 publication of the draft guidance, or any actions taken against products that were not explicitly prioritized in this policy (U.S. Food and Drug Administration, 2020b). This study did not code for illicit retail sales in the U.S. or elsewhere. Additionally, discussions included past, present, future, or hypothetical behaviors and these posts may not represent actual behaviors. Use of paraphrasing may have subtly altered the meaning or interpretation of original quotes. Next, the majority of posts in our sample did not mention any use or purchasing behavior. Users more commonly discussed general topics such as opinions about the policy (e.g., skepticism, negative or positive attitudes, political sentiments about the policy), which were outside the study scope. Last, ENDS-related behaviors around use and purchasing change over time and the conclusions from this study may not apply to other time periods.

## 5 Conclusion

In response to the ENDS prioritized enforcement policy, Reddit posts in our sample commonly discussed switching to other ENDS products not included in this provision of the policy, namely devices with refillable pods/cartridges and open tank systems, and purchasing products from online sources new to the user that still sold restricted flavored products. These findings underscore the benefit of analyzing publicly available social media data from ENDS communities on Reddit to examine potential unintended consequences associated with a policy, and can complement findings from survey and sales data to better understand how the public may have responded to the policy.

## Supplementary Material

Table 1

## Figures and Tables

**TABLE 1:** Mentions of use behaviors based on a sample of Reddit posts about FDA’s ENDS prioritized enforcement policy, January 2, 2020–May 6, 2020^a^.

Use behavior^b^	Number of posts	% (n/N) of posts	
		Use behaviors	Total^c^
Total use behaviors	121	100.0% (121/121)	21.0% (121/576)
Switching behaviors	61	50.4% (61/121)	10.6% (61/576)
ENDS-related switching behavior	56	91.8% (56/61)	9.7% (56/576)
Switching to open tank (mod or unknown)	28	45.9% (28/61)	4.9% (28/576)
Switching to device with refillable pods/cartridges	27	44.3% (27/61)	4.7% (27/576)
Switching to disposable device	5	8.2% (5/61)	0.9% (5/576)
Switching to cartridge-based device with tobacco- or menthol-flavored pods/cartridges	3	4.9% (3/61)	0.5% (3/576)
Switching to zero-nicotine e-liquid	1	1.6% (1/61)	0.2% (1/576)
Other ENDS-related switching behavior^d^	3	4.9% (3/61)	0.5% (3/576)
Switching to non-ENDS tobacco products	4	6.6% (4/61)	0.7% (4/576)
Switching to cannabis	1	1.6% (1/61)	0.2% (1/576)
Mixing own e-liquid (“do-it-yourself” [DIY])	29	24.0% (29/121)	5.0% (29/576)
Cutting back or quitting ENDS	15	12.4% (15/121)	2.6% (15/576)
Continued use of flavored (other than tobacco- or menthol-flavored) cartridge-based ENDS	10	8.3% (10/121)	1.7% (10/576)
Other ENDS use behaviors^e^	10	8.3% (10/121)	1.7% (10/576)
Other tobacco use behaviors^f^	7	5.8% (7/121)	1.2% (7/576)

a Policy announced January 2, 2020 and implemented February 6, 2020.

b Rows are not mutually exclusive.

c Denominator is the final analytic sample of Reddit posts about FDA’s ENDS prioritized enforcement policy.

d Other ENDS-related switching behavior includes mentions of general recommendations to switch to not specified device and product types.

e Other ENDS use behaviors include mentions of stockpiling products not restricted by the policy (e.g., nicotine e-liquids, coils) in preparation for potential new policies or general ENDS use behavior.

f Other tobacco use behaviors include mentions of tobacco use by users who were also discussing past, current, or future ENDS use.

**TABLE 2:** Mentions of purchasing behaviors based on a sample of Reddit posts about FDA’s ENDS prioritized enforcement policy, January 2, 2020–May 6, 2020^a^.

Purchasing behavior^b^	Number of posts	% (n/N) of posts	
		Purchasing behavior	Total^c^
Total purchasing behavior	89	100% (89/89)	15.5% (89/576)
Purchasing from a retailer new to user	29	32.6% (29/89)	5.0% (29/576)
Purchasing from a jurisdiction outside the U.S.	19	21.3% (19/89)	3.3% (19/576)
Purchasing extra other ENDS products^d^	19	21.3% (19/89)	3.3% (19/576)
Purchasing extra flavored (other than tobacco- or menthol-flavored) cartridge-based ENDS	12	13.5% (12/89)	2.1% (12/576)
Purchasing from a direct source (e.g., another user or non-commercial source)	0	0% (0/89)	0% (0/576)
Other purchasing behavior^e^	32	36.0% (32/89)	5.6% (32/576)

a Policy announced January 2, 2020 and implemented February 6, 2020.

b Rows are not mutually exclusive.

c Denominator is the final analytic sample of Reddit posts about FDA’s ENDS prioritized enforcement policy.

d Includes all other ENDS products that were not flavored cartridge-based ENDS.

e Other purchasing behaviors include mentions of purchasing a normal quantity of product(s) not restricted by the policy or general mentions of purchase behaviors.

**TABLE 3:** Mentions of specific flavor^a^ by use and purchasing behavior based on a sample of Reddit posts about FDA’s ENDS prioritized enforcement policy, January 2, 2020–May 6, 2020^b^.

Use and purchasing behavior^c^	Number of posts	% (n/N) of posts	
		Behavior	Total^d^
Total behavior posts that mention specific flavor	39	100% (39/39)	6.8% (39/576)
Use behavior posts that mention a specific flavor	30	24.8% (30/121)^e^	5.2% (30/576)
Switching behavior posts that mention a specific flavor	17	27.9% (17/61)^f^	3.0% (17/576)
Purchasing behavior posts that mention a specific flavor	15	16.9% (15/89)^g^	2.6% (15/576)

a Includes any mention of flavor including flavored (e.g., mint, fruit, dessert), menthol, and tobacco.

b Policy announced January 2, 2020 and implemented February 6, 2020.

c Rows are not mutually exclusive.

d Denominator is the final analytic sample of Reddit posts about FDA’s ENDS prioritized enforcement policy.

e Denominator based on total number of use behavior posts N = 121 (see Table 1).

f Denominator based on total number of switching behavior posts N = 61 (see Table 1).

g Denominator based on total number of purchasing behavior posts N = 89 (see Table 2).

## Data Availability

The raw data supporting the conclusions of this article will be made available by the authors, without undue reservation.

## Footnotes

1 In November 2018, FDA’s commissioner made an initial statement proposing new steps to reduce youth access to ENDS, including reducing access to flavored ENDS (other than mint, menthol, tobacco) (U.S. Food and Drug Administration, 2018). Draft guidance for a prioritized ENDS enforcement policy was published in March 2019 Federal Register (2019).

2 The policy also prioritized enforcement of products for which the manufacturer failed to take adequate measures to prevent minors’ (under age 21) access to and products for which marketing targeted or likely promoted minors’ use.
