# Supplementary material for: Content analysis of conversations on Reddit: reactions to FDA’s ENDS prioritized enforcement policy
Source: Front Commun (Lausanne). Author manuscript; Available in PMC 2025 Jan 28. (PMC11773509; doi:10.3389/fcomm.2024.1348158)
Supplement: Table 1 [file NIHMS2046788-supplement-Table_1.pdf]

Supplementary Material

Search Query:	("flavor policy" OR "flavor policies" OR "FDA-2019-D-0661" OR ((flavor OR flavors) AND (banned OR ban OR bans)))
Subreddits from which data was queried:	reddit.com/r/Canadian_ecigarette<br>reddit.com/r/Cloud_Chasers<br>reddit.com/r/CoilGore<br>reddit.com/r/Coilporn<br>reddit.com/r/DIY_eJuice<br>reddit.com/r/drunkvapes<br>reddit.com/r/E_Cigarette<br>reddit.com/r/ecig_vendors<br>reddit.com/r/ecigclassifieds<br>reddit.com/r/ecr_eu<br>reddit.com/r/ECR_Gaming<br>reddit.com/r/ECR_Plus<br>reddit.com/r/ECRGroupBuy<br>reddit.com/r/ecrmech<br>reddit.com/r/ejuice<br>reddit.com/r/ejuice_reviews<br>reddit.com/r/EJuicePorn<br>reddit.com/r/electronic_cigarette<br>reddit.com/r/ExperiencedVapers<br>reddit.com/r/FreeorCheapEJuice<br>reddit.com/r/hookah<br>reddit.com/r/Innokin<br>reddit.com/r/juul<br>reddit.com/r/OpenPV<br>reddit.com/r/puffbar<br>reddit.com/r/RBA<br>reddit.com/r/shitty_ecr<br>reddit.com/r/SteamHeads<br>reddit.com/r/stopsmoking<br>reddit.com/r/vape_memes<br>reddit.com/r/vapedisposables<br>reddit.com/r/vapeitforward<br>reddit.com/r/VapePorn<br>reddit.com/r/VaperCraft<br>reddit.com/r/VapeRequests<br>reddit.com/r/vapetricks<br>Reddit.com/r/Vaping<br>reddit.com/r/Vaping101
